# Identification of Potential Chemical Substrates as Fuel for Hypoxic Tumors That May Be Linked to Invadopodium Formation in Hypoxia-Induced MDA-MB-231 Breast-Cancer Cell Line

**DOI:** 10.3390/molecules25173876

**Published:** 2020-08-26

**Authors:** Hamad Ali Hamad, Hamid Hammad Enezei, Anmar Alrawas, Noraina Muhamad Zakuan, Nurul Akmaryanti Abdullah, Yoke Kqueen Cheah, Nur Fariesha Md Hashim

**Affiliations:** 1 Department of Biomedical Sciences, Faculty of Medicine and Health Sciences, Universiti Putra Malaysia, Serdang 43300, Malaysia; hamadali91.ha@gmail.com (H.A.H.); anmar.alrawas@gmail.com (A.A.); nurulakmar@upm.edu.my (N.A.A.); ykcheah@upm.edu.my (Y.K.C.); 2 Research and Training Unit, Anbar Cancer Centre, Anbar Health Directorate, Ramadi 31001, Iraq; 3 Department of Oral and Maxillofacial Surgery, Collage of Dentistry, Anbar University, Ramadi 31001, Iraq; drhamed2000@yahoo.com

**Keywords:** cancer invasion, invadopodia, ECM, hypoxia, HIF-1α, phenotype microarray

## Abstract

Hypoxia plays a significant role in solid tumors by the increased expression of hypoxia-inducible factor-1α (HIF-1α), which is known to promote cancer invasion and metastasis. Cancer-cell invasion dynamically begins with the degradation of the extracellular matrix (ECM) via invadopodia formation. The chemical substrates that are utilized by hypoxic cells as fuel to drive invadopodia formation are still not fully understood. Therefore, the aim of the study was to maintain MDA-MB-231 cells under hypoxia conditions to allow cells to form a large number of invadopodia as a model, followed by identifying their nutrient utilization. The results of the study revealed an increase in the number of cells forming invadopodia under hypoxia conditions. Moreover, Western blot analysis confirmed that essential proteins for hypoxia and invadopodia, including HIF-1α, vascular endothelial growth factor (VEGF), metallopeptidase-2 (MMP-2), and Rho guanine nucleotide exchange factor 7 (β-PIX), significantly increased under hypoxia. Interestingly, phenotype microarray showed that only 11 chemical substrates from 367 types of substrates were significantly metabolized in hypoxia compared to in normoxia. This is thought to be fuel for hypoxia to drive the invasion process. In conclusion, we found 11 chemical substrates that could have potential energy sources for hypoxia-induced invadopodia formation of these cells. This may in part be a target in the hypoxic tumor and invadopodia formation. Additionally, these findings can be used as potential carrier targets in cancer-drug discovery, such as the usage of dextrin.

## 1. Introduction

Hypoxia is observed in the majority of solid tumors. In solid tumors, aggressiveness, angiogenic activity, and tumor progression (enhanced due to low-oxygen) exist in most if not all malignant cancers that are known to resist chemotherapy and radiotherapy. Studies reported that hypoxia is a driving force for breast-cancer progression [1,2]. Previous studies showed that hypoxia-inducible factor-1α (HIF-1α), a master regulator protein in hypoxia, plays an essential role in cancer invasion and metastasis, and evidence suggests that high HIF-1α expression displays poor prognosis for cancer patients, particularly those with breast cancer [3]. Recent reports approved that HIF-1α promotes invadopodia formation in hypoxia conditions through well-known signaling and proteins, including lysophosphatidic acid 1-epidermal growth factor receptor (LPA1–EGFR) signaling axis, and through the upregulation of cysteine and glycine-rich protein 2 (CSRP2) invadopodium actin-bundling protein [4,5].

Hypoxia in solid tumors can alter cancer-cell metabolism and contributes to therapy resistance by inducing cell quiescence [6]. Adaptation to hypoxia needs various genetic and biochemical responses that regulate each other to survive and metastasize [7]. The adaptation of tumor cells to hypoxia is believed to be the main driver for the selection of more invasive and therapy-resistant phenotypes [8]. This is because of HIF-1α, which is known to induce multidrug resistance in many types of cancer, such as breast and colon cancers [9].

HIF-1α can also promote metastasis, which is a critical step in all malignant cancers in which cancer cells spread away from the primary tumor to distant organs. The majority of cancer mortality is due to metastasis [10]. It occurs when malignant cells detach from the original tumor to invade surrounding tissue and blood vessels, where they proliferate and grow to establish a secondary tumor [11,12]. Malignant-cancer cells can invade the extracellular matrix (ECM) through fingerlike protrusions called invadopodia. Previous and recent reports have found that invadopodium formation is critical for ECM degradation and invasion in metastasis [13,14].

Invadopodia are specialized actin-rich membrane protrusions that contain actin filaments, and they are formed at the ventral surface of malignant cells, such as MDA-MB-231 breast-carcinoma cells [15]. The MDA-MB-231 cell line is widely used to model the metastatic stage of triple-negative breast cancer (TNBC). Since this cell line lacks progesterone receptors (PRs), estrogen receptors (ERs), and human epidermal growth factor receptor-2 (HER-2), this cell line represents a good model for TNBC. A study conducted by Kang et al. (2003) [16] showed that MDA-MB-231 cells have an invasive potential in vitro; when implanted in vivo, they produced xenografts that spontaneously metastasized to lymph nodes.

The ability of malignant cells to form invadopodia usually depends on their invasiveness potential [17,18,19]. Invadopodia revealed multiple core neoplastic roles associated with cancer malignancy; for example, invasiveness requires actin-regulating proteins, such as actin-related proteins 2/3 (Arp2/3), cortactin, Wiskott–Aldrich syndrome protein-interacting protein (WIP), and proteolysis; these signals are required for metallopeptidase-2/metallopeptidase-9 (MMP-2/MMP-9) and membrane Type 1-matrix metalloproteinase (MT1-MMP) to degrade the ECM in the metastasis process [18,20]. Therefore, inhibiting the function of invadopodia may in part be a suppressor for metastatic progression [21].

In the current study, one area that is yet to be explored is carbon-source utilization that MDA-MB-231 breast-cancer cells can consume as nutrient providers for hypoxia conditions to drive the formation of invadopodia. A recent report established that improved cancer therapy may be achieved by exploring the nutrient utilization of tumor cells [22]. Thus, to expand on the study on hypoxia-induced invadopodium formation in MDA-MB-231 cells, we designed a model to maintain cancer cells under hypoxia by using a hypoxia chamber (1% O_2_) and 0.5 mM of dimethyloxalylglycine (DMOG), a chemical compound that works as an HIF hydroxylase inhibitor that is widely used to mimic hypoxia conditions in order to use phenotype microarrays for mammalian cells.

## 2. Results

### 2.1. Hypoxia Induction Using Hypoxia Chamber and DMOG Treatment to Visualize and Quantify Invadopodia Formation

**Figure 1:**
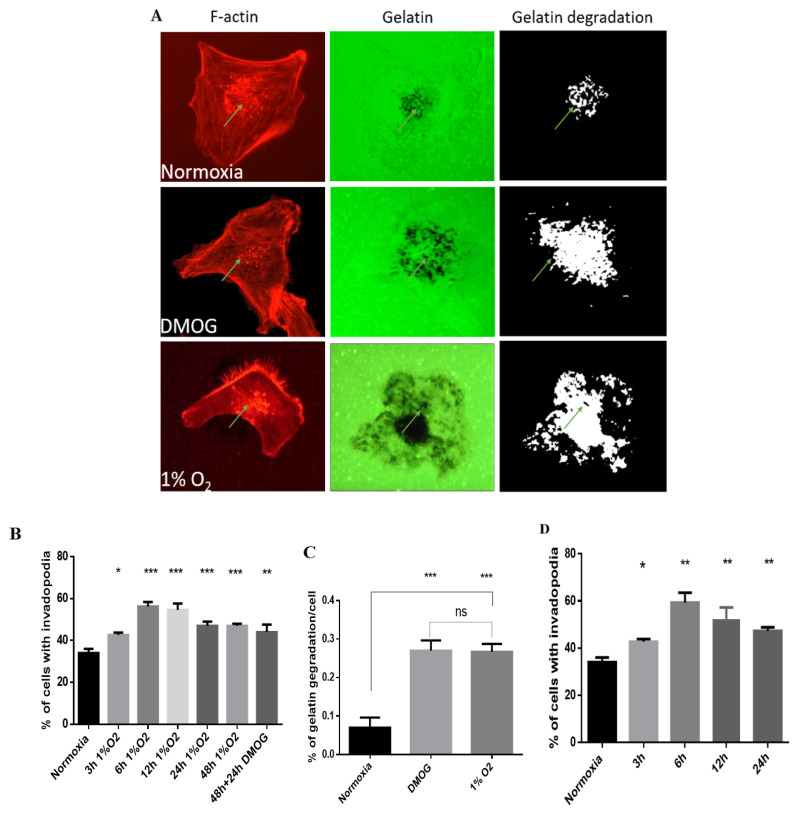
Hypoxia increases invadopodium formation and gelatin degradation in MDA-MB-231 cells. (**A**) Invadopodia-positive MDA-MB-231 cells stimulated with hypoxia by hypoxia chamber (1% O_2_) and dimethyloxalylglycine (DMOG) treatment for 6 h, then seeded on gelatin-coated coverslips for 3 h, followed by fixing and staining for F-actin by rhodamine–phalloidin. Arrows, invadopodium formation. Gelatin degradation measured by ImageJ software; scale bar = 10 μm. (**B**) Cells with invadopodia counted for presence of actin puncta that colocalized with gelatin degradation, quantified in time-dependent manner compared to in normoxia. (**C**) Quantification of gelatin degradation per cell in 3 different conditions. (**D**) Cells treated with DMOG alone for specific incubation time compared to normoxia; cells counted as in (**B**). Shown data, mean of 100 cells each from three independent experiments. Statistical significance analyzed using one-way ANOVA by GraphPad Prism 6; All data are the mean ± S.E.M of three independent experiments. * *p* < 0.05, ** *p* < 0.01 and *** *p* < 0.001, which is significantly different from other groups.

Invadopodia formation in the MDA-MB-231 breast-cancer cell line in normoxia, dimethyloxalylglycine (DMOG), and the hypoxia chamber (1% O_2_, 5% CO_2_, and balanced with N_2_) was examined after seeding hypoxic or nonhypoxic cells on gelatin-coated coverslips. One of the key components of invadopodia, actin, was stained, and it localized at the center of the cells (Figure 1A). Representative images of invadopodia formation in DMOG-treated cells and the hypoxia chamber showed that the gelatin-degradation area was increased in hypoxia compared to in normoxia (Figure 1A). Only about 30%–40% of total cell populations form invadopodia under normal conditions (Figure 1A). Next, we determined the number of cells forming invadopodia at different incubation times under hypoxia conditions as a model in order to design the phenotype-microarray experiment. Results revealed that the number of MDA-MB-231 cells forming invadopodia was significantly high for cells incubated with the hypoxia chamber for (3–48) h compared to in normoxia (Figure 1B). Furthermore, MDA-MB-231 cells incubated for 48 h in a hypoxia chamber, followed by 24 h with 0.5 mM DMOG, showed higher levels of invadopodia formation compared to normoxic MDA-MB-231 cells (Figure 1B). We also confirmed that the gelatin-degradation area in the hypoxia chamber and DMOG treatment had the same gelatin-degradation effect on MDA-MB-231 cells, and a significant effect was observed when compared to that in normoxia (Figure 1C). Additionally, we investigated the effect of 0.5 mM DMOG alone on invadopodia formation for (3–24) h, respectively. Results showed a significant increase in cells treated with DMOG with invadopodia compared to those in normoxia (Figure 1D). Therefore, this result gave preliminary data to maintain MDA-MB-231 cells in a highly invasive condition that could lead us to investigate carbon- and nitrogen-source utilization as fuel by using a phenotype microarray compared to normoxia.

### 2.2. Expression of HIF-1α and VEGF in MDA-MB-231 Cells

**Figure 2:**
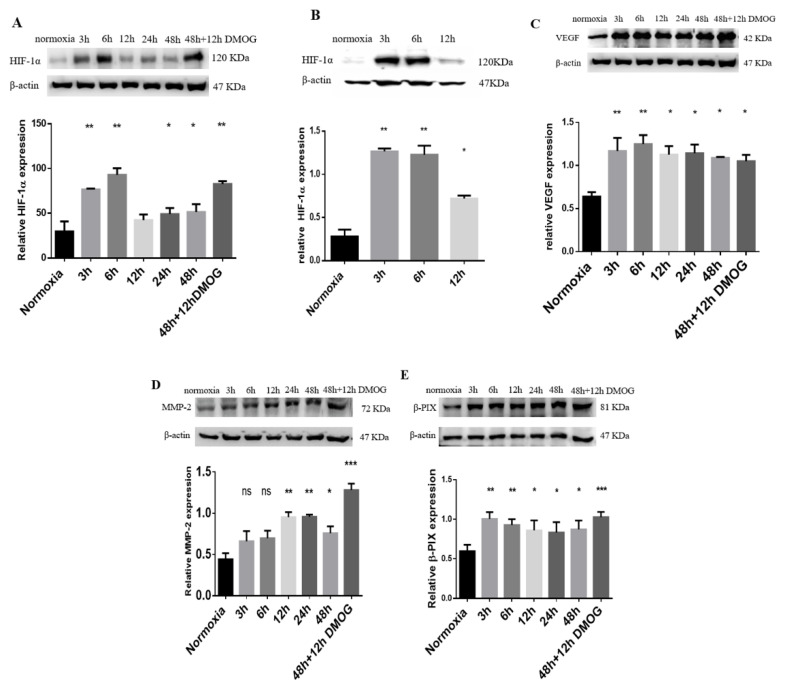
Hypoxia condition increases expression of essential proteins for hypoxia and invadopodium formation. MDA-MB-231 cells incubated in hypoxia chamber alone for 3 to 48 h, and with addition of DMOG for 12 h. (**A**) Hypoxic and normoxic cell lysates probed for hypoxia-inducible factor-1α (HIF-1α) expression. (**B**) Cells treated with 0.5 mM DMOG for (3, 6, 12) h to detect HIF-1α expression compared to in normoxia (control). (**C**) Hypoxic and normoxic cell lysates probed for vascular endothelial growth factor (VEGF) as positive control for HIF-1α expression. (**D**) Hypoxic and normoxic cell lysates probed for expression of MMP-2 and re-probed for expression of Rho guanine nucleotide exchange factor 7 (β-PIX) (**E**). β-actin used as loading control in all protein-expression experiments. Densitometric analysis performed by ImageJ software and GraphPad Prism 6 using one-way ANOVA. All data are the mean ± S.E.M of three independent experiments except MMP-2, which was repeated twice. * *p* < 0.05, ** *p* < 0.01 and *** *p* < 0.001, which is significantly different from other groups.

To confirm the hypoxia-induced invadopodium-formation model, we investigated the effect of HIF-1α expression that increases the invasiveness of breast-cancer cells [17]. Western blot analysis showed that HIF-1α was absent in normoxic MDA-MB-231 cells, while it was expressed in hypoxia. HIF-1α expression was significantly increased in cells that were incubated in a hypoxia chamber (1% O_2_) for (3, 6, 24, and 48) h compared to in normoxia. However, at 12 h incubation, the expression level of HIF-1α was relatively low, yet it was still higher than that in normoxic cells (Figure 2A). Moreover, cells incubated for 48 h in a hypoxia chamber were further incubated for 12 h after adding 0.5 mM of DMOG; surprisingly, a significant expression of HIF-1α was observed (Figure 2A). This finding was essential in order to perform the phenotype-microarray experiment. Additionally, we confirmed that 0.5 mM DMOG alone significantly increased HIF-1α expression (Figure 2B). Additionally, we used vascular endothelial growth factor (VEGF) as a positive control to validate HIF-1α expression. Results showed that VEGF significantly increased in 3 to 48 h plus 12 h, respectively (Figure 2C).

### 2.3. MMP-2 and β-PIX Expression Levels in Hypoxia-Induced MDA-MB-231 Cells

Previous reports stated that MMP-2 and Rho guanine nucleotide exchange factor 7 (β-PIX) are essential for invadopodia formation in MDA-MB-231 cells [23,24]. Here, we investigated these two proteins under hypoxia conditions to confirm that the molecular component of invadopodia increases under hypoxia. Western blot analysis showed that MMP-2 expression significantly increased in cells that were incubated in the hypoxia chamber for (12, 24, 48, and 48) h, plus 12 h with the addition of 0.5 mM DMOG (Figure 2D). Additionally, β-PIX expression significantly increased in cells exposed to hypoxia for (3–48) h, respectively, and a further significant increase was detected in cells when incubating for 48 h in a hypoxia chamber, plus 12 h treated with 0.5 mM DMOG (Figure 2E). Results confirmed that the model increased the expression of molecular components of invadopodium formation under hypoxia conditions. (Figure 2C).

### 2.4. Phenotypic Characterization of Hypoxia-Induced MDA-MB-231 Cells

**Figure 3:**
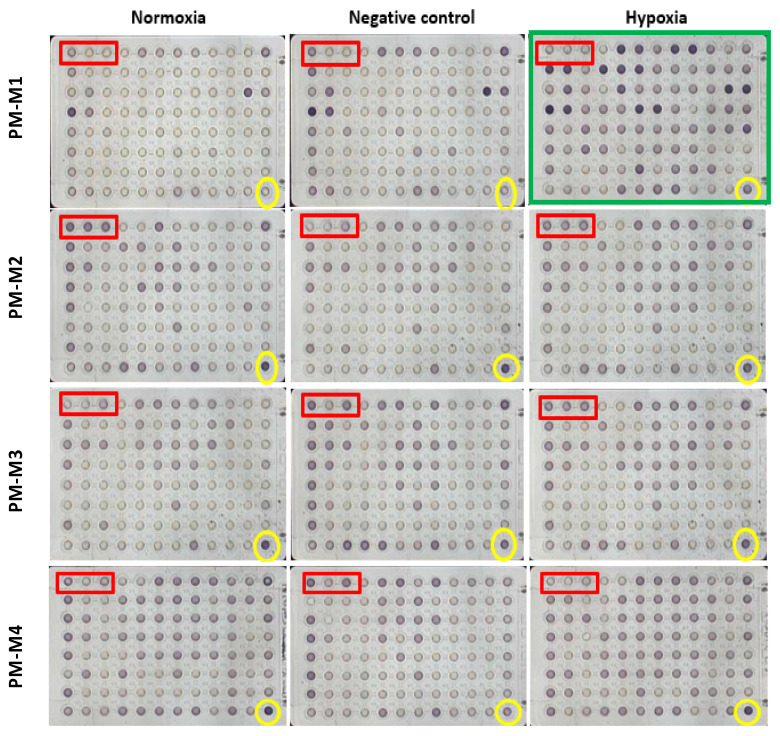
Phenotype-microarray plates containing hypoxia-/non-hypoxia-induced MDA-MB-231 cells. Phenotype-microarray (PM)-M1 through PM-M4 plates, containing 367 chemical substrates in each well, may have provided energy. Plates incubated for 48 h in a hypoxia chamber or in a humidified atmosphere of 95% air 5% CO_2_ in an incubator to allow cells to utilize provided nutrients in the media. This was followed by allowing cells to undergo a transition in their metabolism to use various chemical substrates precoated in each well during an additional 24 h incubation in the OmniLog reader after 10 μL of Biolog Redox Dye Mix (MA dye) was added into each well. The OmniLog reader recorded the color formation of MA dye every 15 min. The OmniLog reader photographed plates for 24 h every 15 min.

**Figure 4:**
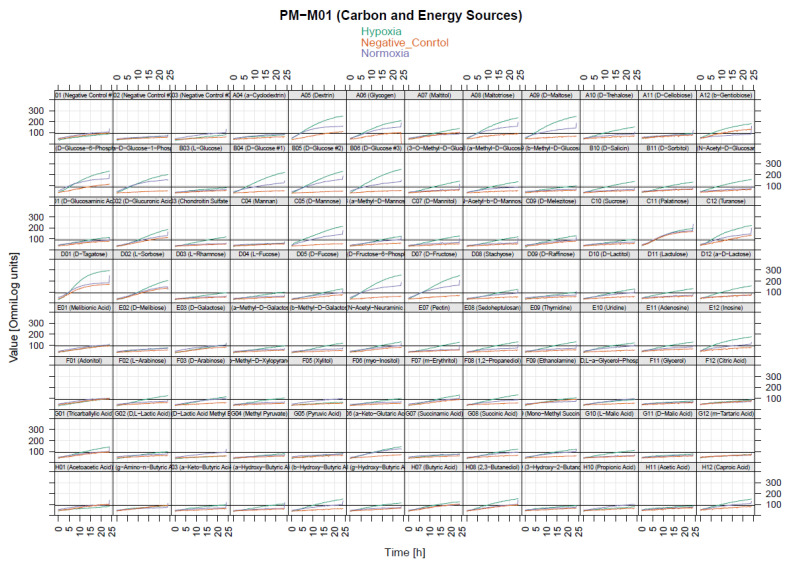
Metabolic fingerprint of phenotype microarray in MDA-MB-231 cell line. Graphical depiction of PM-M1 plate from (0–24) h in OmniLog reader; 96-well plate representation in each plate. Each well represents three groups: hypoxia, normoxia (control), and negative control (without cells). Shown is time on x axis versus OmniLog value on y axis. PM-M1 plate shows that hypoxic cell group utilized many substrates as energy, as indicated in green line.

We hypothesized that hypoxia utilizes more chemical substrates as fuel for cellular activity, such as the invasion process. To investigate the preferences of chemical substrates under hypoxia conditions in MDA-MB-231 cells, a phenotype microarray for mammalian cells was employed to understand why cancer-cell invasion was promoted under hypoxia conditions, particularly by the increase in the level of invadopodium formation, by monitoring the levels of carbon- and nitrogen-source utilization. Phenotype microarray comes in four 96-well plates precoated with 367 types of chemical substrates. A high number of cells forming invadopodia were maintained under hypoxia conditions. We incubated phenotype-microarray (PM) plates containing the cells in the hypoxia chamber for 48 h in order to allow the cells to consume the residual nutrient provided in the media. Following that, cells were treated with 0.5 mM DMOG; at that time, the cells started to utilize the chemical substrates provided in each well. Interestingly, results showed that several substrates were utilized in hypoxic MDA-MB-231, specifically in the PM-M1 plate that contained primary carbohydrate and carboxylate substrates as a single nutrient source for their survival by turning purple as energy (Figure 3). Furthermore, to visualize the data in three groups, the OPM package that is used by R software language was used for analysis. Interestingly, analysis using the OPM package revealed that 21 types of substrates were detected in the hypoxia group in the PM-M1 plate as preliminary data (Figure 4). However, PM-M2, PM-M3, and PM-M4 that contain L-amino acids, and most dipeptide combinations, exhibited inhibition in cell survival, with no changes in hypoxia compared to normal conditions (Supplementary Data 1). This could be due to MDA-MB-231 cells not utilizing the biochemical substrates or the dipeptides being toxic to the cells.

### 2.5. Identification of Significant Chemical Substrates in PM-M1 Plate

**Figure 5:**
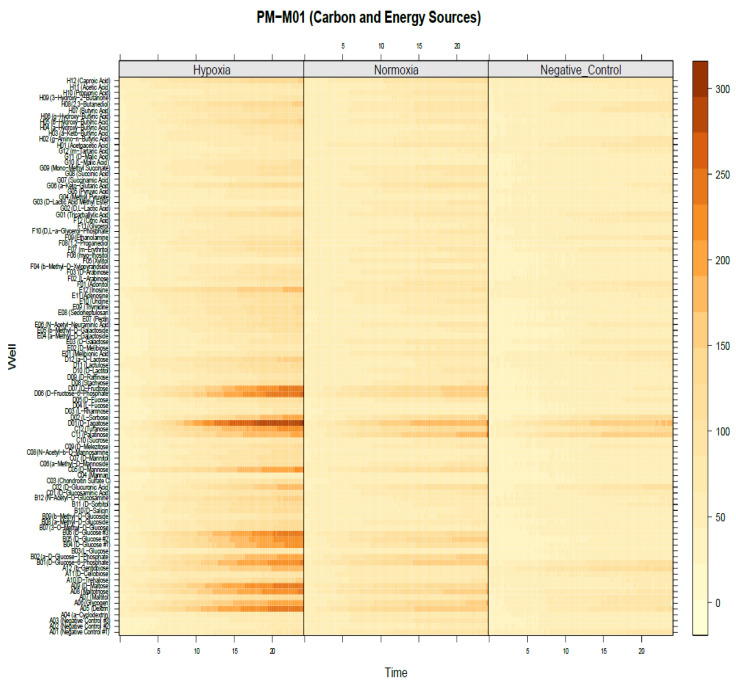
Heat map shows high energy production from carbon sources in hypoxia group in PM-M1. Time shown on the x-axis versus substrate names in each well on the y-axis. The Hypoxia group demonstrated high intensity (brown), which reflects the amount of nicotinamide adenine dinucleotide (NADH) energy that was utilized by MDA-MB-231 cells from chemical substrates. The Normoxia group showed that NADH production in MDA-MB-231 cells was less than that in the hypoxia group. The Negative control (without cells) group, composed of only medium plus DMOG without cells, revealed some abiotic reaction between chemical substrates and DMOG treatment. Heat map visualized and analyzed with OPM package.

**Figure 6:**
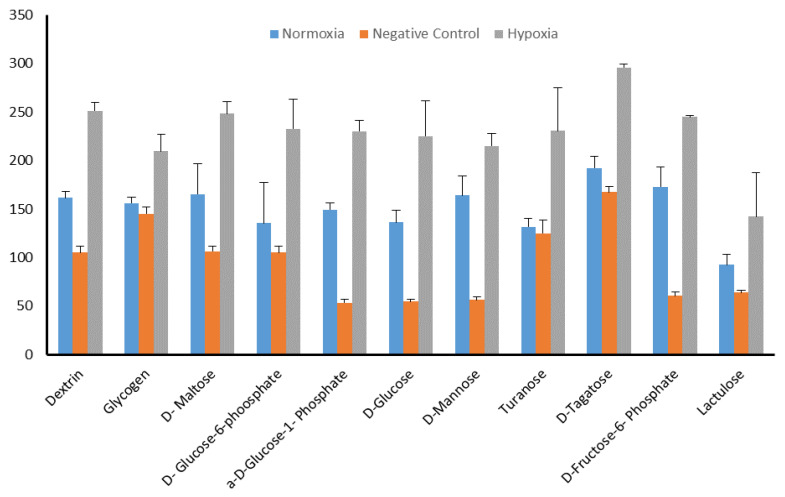
Chemical-substrate utilization after 24 h incubation with MA mix dye in OmniLog reader. Referring to heat-map data, only 11 types of chemical substrates were found that were significantly increased in hypoxia compared to normoxia (control) and negative control (without cells). Graph visualized by Excel, and analysis performed by GraphPad Prism 6 using one-way ANOVA. *p*-value < 0.05 considered significant. The Hypoxia group, compared to negative control group (without cells), was significant in 11 substrates. Y axis shows the OmniLog value (color density units) that reflected metabolism intensity in cells.

**Figure 7:**
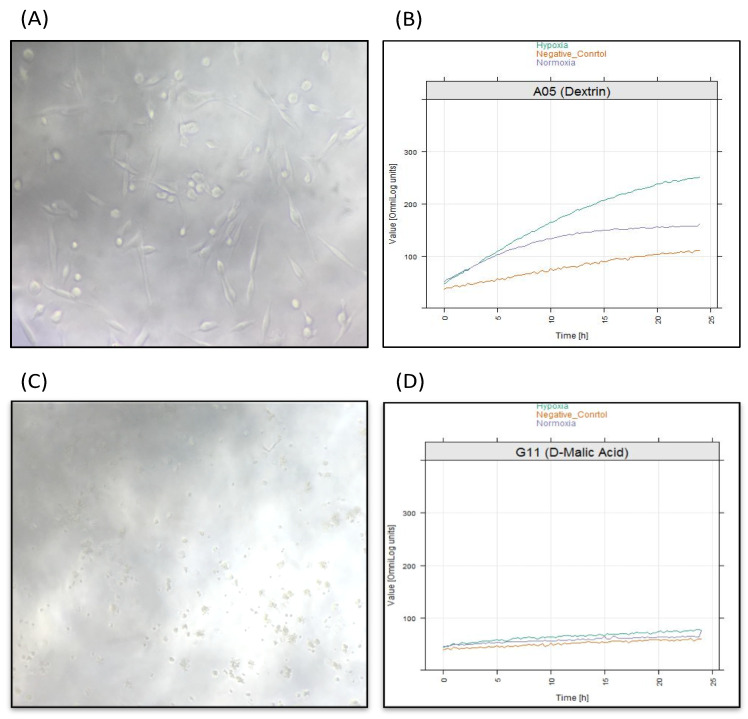
MDA-MB-231 cells under different chemical substrates. (**A**) Microscopic image of MDA-MB-231 cells in a well containing dextrin after 72 h of incubation shows cells were surviving well under hypoxia conditions. (**B**) Quantification of energy levels that were produced by dextrin utilization in hypoxia and normoxia conditions. (**C**) Microscopic image of MDA-MB-231 cells in well containing d-malic acid after 72 h incubation shows cells were killed under hypoxia conditions. (**D**) Quantification of energy levels produced by d-malic acid utilization in hypoxia and normoxia conditions. Scale bar = 100 µm.

To identify the significance in chemical substrates that were utilized under the hypoxia condition in PM-M1, we used heat-map analysis provided by the OPM package. Results showed that several chemical substrates were utilized and increased in dye reduction that presented high potency of energy under hypoxia compared to under normoxia (Figure 5). Further analysis was also done to identify the specific substrate under the hypoxia condition. Interestingly, only 11 types of chemical substrate were significant including dextrin, glycogen, d-maltose, d-glucose-6-phosphate, a-d-glucose-1-phosphate, d-glucose, d-mannose, turanose, d-tagatose, d-fructose-6-phosphate, and lactulose (Figure 6). These substrates could be energy sources as fuel for invadopodium formation under hypoxia conditions in MDA-MB-231 cells. For example, dextrin substrates showed that, after 72 h in hypoxia conditions, MDA-MB-231 cells were still viable (Figure 7A,B), while d-malic acid killed the cells (Figure 7C,D).

## 3. Discussion

The present study underlined the characterization of invadopodia under hypoxia conditions to find essential chemical-substrate utilization during hypoxia that may correlate with the invasion process, particularly invadopodium formation in the MDA-MB-231 breast-cancer cell line. Results confirmed that hypoxia conditions significantly promote invadopodium formation and found 11 substrates that maintained cell survivability under hypoxia and may be related to invadopodium fuel. Related reports showed that invadopodium formation was enhanced in hypoxia through CSRP2 and WIP expressions in MDA-MB-231 cells [25,26]. Additionally, our findings showed that invadopodia are formed in the center of the cells, which is consistent with a previous report [27].

Moreover, the expression level of HIF-1α significantly increased under hypoxia conditions using a hypoxia chamber (1% O_2_). These results were validated and confirmed by the expression of VEGF as a positive control for HIF-1α. VEGF is not only a positive control for HIF-1α, but also a well-known marker for angiogenesis and tumor growth [28]. This result was consistently seen in cells treated with 0.5 mM DMOG under normoxia conditions. Similar reports concluded that highly invasive cancer cells respond to hypoxia by the stabilization of HIF-1α, which can alter the expressions of many proteins required for invadopodium formation [16,29]. In addition, previous reports using MDA-MB-231 cells revealed that overexpression of HIF-1α increases invadopodium formation and enhances ECM degradation compared to normoxia [30,31]. Thus, we tested the expression level of β-PIX and MMP-2, as these proteins play a critical role in invadopodium formation [23,24]. Our findings showed that the expression level of MMP-2 and β-PIX under hypoxia significantly increased compared to normoxia (control). A number of studies reported that MMP families, including MMP-2, are enriched at invadopodia and required for cancer-cell invasion [32,33,34]. Similar findings in MDA-MB-231 cells showed that overexpression of β-PIX can induce invadopodium formation via increased expression of HIF-1α [24].

These extensive significant results gave us an idea to design a model for the phenotype-microarray experiment, and to maintain the cancer cells forming a high number of invadopodia as soon as the cells were incubated in hypoxia chamber for 48 h, followed by 12 h of treatment with 0.5 mM DMOG, which is the lowest concentration to avoid the abiotic reaction between DMOG and chemical substrates precoated in each well of the PM plates. The hypoxia chamber and DMOG were used together to keep cancer cells under a hypoxia condition. Furthermore, the hypoxia chamber alone was used to allow cells to utilize the residual nutrients provided in the media. After 48 h, cells were then treated with 0.5 mM DMOG with the same medium mixed with MA dye to switch to new nutrients provided in each well for 24 h in the OmniLog reader. The reader photographed the plates every 15 min for 24 h to provide sufficient time to utilize the chemical substrates provided in each well as a single source for survival.

Strikingly, the PM findings showed that only 11 types of carbon substrates, namely, dextrin, glycogen, d-maltose, d-glucose-6-phosphate, a-d-glucose-1-phosphate, d-glucose, d-mannose, turanose, d-tagatose, d-fructose-6-phosphate, and lactulose, were significantly utilized in hypoxia compared to normoxia. Thus, these 11 types of carbon substrates could be the fuel that is responsible for driving invadopodium formation under hypoxia. A recent related report revealed that glucose utilization is essential for cell metabolism and HIF-1α expression to generate energy [35]. Our results showed that glucose is significantly utilized under hypoxia conditions compared to in normoxia. This is in line with a previous study that reported that glucose demand increases in hypoxia conditions due to high expressions of glucose transporter 1 (GLUT1) and glucose transporter 2 (GLUT2), which are known to transport a glucose molecule that is regulated by HIF-1α [36]. Therefore, glucose utilization could be a reason behind the increase in invadopodium formation in hypoxia-induced MDA-MB-231 cells.

A recent study concluded that cell metabolism and carbohydrate availability, especially pyruvate, are involved in fueling invadopodium formation and activity [37]. Similarly, glucose is considered a fundamental substrate for energy in the majority of cancers [38]. However, our results showed other chemical substrates are utilized profusely under hypoxia-induced MDA-MB-231 cells as an energy provider, for example, dextrin. We speculate that these types of substrates may be required for invadopodium formation because changes in nutrition consumption, particularly carbohydrates, appear to be involved in becoming fuel for invadopodium formation [37]. In contrast, these chemical substrates need to be individually tested to explore their role in the formation of invadopodia to uncover the energy during invadopodium formation.

On the other hand, the mentioned significant chemical substrates, such as dextrin, can potentially be a drug carrier targeting invadopodium formation under hypoxia. Our findings showed that dextrin was significantly more metabolized in hypoxia than in normoxia. Additionally, the morphology of MDA-MB-231 cells survived after 72 h incubation in hypoxia. However, the d-malic acid substrate that was tested in the PM-M1 plate inhibited cell survival. Therefore, d-malic acid can be used as an invadopodium inhibitor supported by the medium in low concentrations (in vitro). Results provided evidence for using dextrin as a drug carrier to enhance the effectiveness of cancer therapy against hypoxia and cancer invasion; this could be a novel factor to focus on in hypoxia studies. A similar report used dextrin as a drug carrier in nanoparticle applications to attack a specific target in cancer therapeutics in normal conditions [39]. Dextrin belongs to a group of low-molecular-weight carbohydrates. It is produced by the hydrolysis of glycogen or starch that is widely used to enhance the effectiveness of anticancer therapy [40]. In therapeutic-cancer-drug carriers, there is a developing pattern where malignant-cancer drugs are being incorporated into intelligent drug carriers in order to reduce non-targetable attachment against normal organs.

Thus, these findings suggest the further exploration and testing of dextrin or even other significant substrates to use them as a drug carrier to enhance the effectiveness of chemotherapy. This may improve the selectivity of the drugs to target the hypoxia region, cancer invasion, and metastasis. Taken together, our investigation into what invadopodia fuel is in a hypoxia background is still ongoing, and what we found are preliminary findings on invasion energy. Those chemical substrates as drug carriers are potential candidates to enhance the effectiveness of chemotherapy.

## 4. Materials and Methods

### 4.1. Cell Culture

MDA-MB-231 breast-cancer cell line was obtained from American Type Culture Collection (ATCC, Manassas, VA, USA). Cells were cultured in Dulbecco’s Modified Eagle’s Medium (DMEM), high-glucose medium purchased from Gibco (Gaithersburg, MD, USA) containing FBS 10% and 1% penicillin/streptomycin. Cells were maintained in a humidified atmosphere of 95% O_2_ and 5% CO_2_ incubator at 37 °C for normal conditions. For hypoxia conditions, cells were incubated for a specific amount of time in a hypoxia chamber (Stemcell Technologies catalog #27310) with 1% O_2_, 5% CO_2,_ and balanced with N_2_, and then treated with DMOG (purchased from Sigma) at a final concentration of 0.5 mM to mimic hypoxia. Cells with low passage numbers were used in all experiments in order to get accurate results.

### 4.2. Preparation of Gelatin-Coated Coverslips for Invadopodium Assay

To coat the glass coverslip, 12 mm round coverslips were cleaned using alcohol for 90 min at 70% alcohol, and 30 min at 96% alcohol. Then, coverslips were placed on a filter paper to dry in a biosafety cabinet. After that, Oregon Green^®^ 488 Conjugate gelatin from pigskin (Invitrogen, Carlsbad, CA, USA) was used to coat glass coverslips prior to seeding cells. The protocol was carried out according to Enderling et al. (2008) with slight modification [41]. Coverslips were placed onto parafilm in the dark for 10 min with 30 μL of 0.2 mg/mL gelatin-diluted PBS having 2% sucrose. Then, coverslips were placed onto another parafilm containing 100 μL of 0.5% glutaraldehyde. After 15 min of incubation in the dark, coverslips were moved to a 24-well plate with the gelatin side up. After three times washing with PBS, 1 mL of 5 mg/mL sodium borohydride was added into each well. After 3 min, sodium borohydride was discarded, and washed with PBS and 70% alcohol, each three times. Following coverslips drying on the bench in the dark, 1 mL of DMEM was added onto each well. Prior to seeding cells, cells were cultured overnight in a 6-well plate, then placed in the hypoxia chamber or treated with 0.5 mM DMOG for a specific period of time. Cells were re-trypsinized and counted; then, 2 × 104 cells/mL of cells were seeded into each well of a 24-well plate containing gelatin-coated coverslips, followed by 3 h of incubation. Following that, the media were discarded, and coverslips were gently washed with PBS and fixed with 4% paraformaldehyde for 20 min. Then, cells were washed with PBS three times and permeabilized with 500 μL of 0.2% Triton X-100 into each well for 5 min. Cells were then stained with rhodamine–phalloidin (1:100) diluted in PBS, containing 3% BSA for 1 h; then, the gelatin-coated coverslips were incubated with Hoechst (1:1000) diluted with PBS for 5 min. The gelatin-coated coverslips were then washed three times with ddH_2_O and transferred to slides containing a drop of antifade, a mounting solution. For invadopodium quantification, 100 cells were randomly selected from each coverslip under a fluorescence microscope (Leica, Germany); the mean percentage of cells forming invadopodia was calculated. ImageJ was used to measure the area of matrix degradation per cell of invadopodium-forming cells in different conditions according to a previous report [42].

### 4.3. Protein Extraction and Western Blot Analysis

Whole protein was extracted from MDA-MB-231 cells. Cells were grown in T25 flasks; when confluency reached 80%, cells were exposed to a hypoxia chamber of 1% O_2_ and/or treated with 0.5 mM DMOG for the indicated time. The cell lysate was done quickly to save HIF-1α from degradation by adding 200 μL of RIPA buffer mixed with protease inhibitor (100:1) on the ice plate. Following that, a PCA kit (Merck, Darmstadt, Germany) was used to determine the concentration of the protein. Each sample contained 20 μg of total protein, which was then loaded and separated by 10% SDS-PAGE and blotted on a PVDF membrane. To block the membranes, 5% of BSA with tris buffered saline–Tween 20 (TBS-T) was added for one hour in a shaker, and then incubated overnight at 4 °C on the shaker with the following primary antibodies and dilutions: HIF-1α (1:1000, BD Biosciences, Franklin Lakes, New Jersey, US), MMP-2, VEGF, Cool/β-PIX (1:1000, Cell Signaling Technology, Danvers, MA, USA), and β-actin (1:10000, Santa Cruz, CA, USA); antibodies were diluted with TBS-T buffer containing 5% BSA. Following that, the primary antibody was discarded and washed 3 times with TBS-T each time for 10 min. Membranes were then incubated with HRP-conjugated secondary antibodies for one hour (1:10000 for β-actin and 1:1000 for other antibodies). Then, PVDF membranes were washed with TBS-T three times, each time for 10 min, and viewed under chemiluminescence using a gel-documentation system (Vilber Lourmat, Vilber, Marne-la-Vallée, France) after incubating membranes with enhanced chemiluminescent reagents (ECL). Band quantification was measured using ImageJ software.

### 4.4. Phenotype Microarray (PM) for Mammalian Cells

The PM principle is a cell-based assay used to determine around 1400 phenotypic metabolisms of mammalian surviving cells. PM provides 96-well microplates precoated with various types of carbon and nitrogen sources in the bottoms, including fatty acids, carboxylic acids, amino acids, bi-amino acids, fatty acids, ketones, carbohydrates/starches, and alcohols [43]. Furthermore, PM is characterized by high throughput that tests numerous cellular phenotypes through an OmniLog TM reader. It monitors the response of living cells over a time course to various types of carbon and nitrogen sources [38]. Cells in the OmniLog reader could produce nicotinamide adenine dinucleotide (NADH) in various wells. Mammalian cells simultaneously reduced redox dye formation and increased purple levels that were measured and recorded every 15 min for 24 h in the OmniLog reader. Purple intensity reflected the amount of energy produced by cells in each well [38]. The experiment was carried out according to the phenotype-microarray protocol. Low passage numbers of MDA-MB-231 cells were grown in a T75 flask in normal conditions until 90% confluence. After twice washing cells with d-PBS, 2 mL of trypsin was added into each flask. Then, the cell suspension was collected and once washed with d-PBS using 4 °C centrifugation. After that, d-PBS was discarded, and 3 mL of IF-M1 medium containing 1% penicillin and streptomycin, 0.3 mM glutamine, and 5% FBS was added to the pellet. The pellet was then resuspended by pipetting up and down several times. Cell viability was ~97% using trypan blue in all experiments. Cells were seeded into PM plates; each well contained 2 × 10^4^ cells/well in 50 μL of the medium. The number of cells and incubation time were optimized prior to the actual experiment to ensure that the cells utilized residual nutrients provided with the IF-MI medium before they adapted and switched to the saturating single-chemical substrates precoated in each well of the PM plates. Following that, normoxia plates were incubated in a humidified atmosphere of 95% air and 5% CO_2_ in the incubator for 48 h; then, hypoxia-group and negative-control plates were incubated for 48 h in a hypoxia chamber and placed into 37 °C. The hypoxia chamber was refilled with mixed gas every 12 h to keep cells under the hypoxia condition. The negative-control group contained only Biolog IF-M1 medium. After incubation for 48 h, 10 μL of Biolog Redox Dye Mix (MA dye) was added into each well of the normoxia group. Then, 10 μL of MA dye mixed with DMOG was added. The total concentration of DMOG in each well was 0.5 mM in the hypoxia and negative-control groups. After that, all PM plates were sealed with adhesive film and placed in the OmniLog incubator reader set at 37 °C using standard operating conditions, which collected data every 15 min over a 24 h period. Tetrazolium reduction was kinetically measured by the OmniLog system in which the kinetic readout of color formation was obtained, and tetrazolium-reduction rate was determined.

### 4.5. Statistical Analysis

All performed experiments were analyzed using GraphPad Prism (GraphPad, CA, USA), and expressed as mean values with SD of three separate experiments unless otherwise indicated. Statistical significance was calculated by one-way ANOVA or Student′s *t-*test with a *p* value < 0.05. With regards to phenotype microarrays, data were analyzed using a special technique for visualization and analysis. High-throughput phenotypic data were extracted as raw kinetic profiles using OM_FM software (Biolog Inc., NY, USA, 2009). Then, data were inserted into the OPM package in R software as a CSV-file type for efficient and meaningful statistical analysis and visualization. Analysis steps were carried out using the OPM R package and divided into three stages: grouping, normalization, and affect identification [44,45]. Results were plotted array-wise to reveal the metabolic profile using OmniLog units (OLUs) over time (h).

## 5. Conclusions

In this study, the carbon sources that were greedily consumed under hypoxia hold great potential for developing targeted therapy specifically for cancer cells. In this study, we found 11 chemical substrates that could be considered as fuel for hypoxia-induced invadopodium formation in MDA-MB-231 cells. One potential candidate is dextrin, a substrate that may drive invadopodia under hypoxia conditions. The findings propose an opportunity to use some types of carbon sources, like dextrin, as drug carriers to enhance the effectiveness of chemotherapy in combatting tumor hypoxia and cancer-cell invasion. Apart from that, other chemical substrates can be used as inhibitors of invadopodium formation, such as a low concentration of d-malic acid.

## Supplementary Materials

The following are available online, Data 1. The metabolic fingerprint of phenotype microarray in MDA-MB-231 cell line. Graphical depiction of PM-M2 to PM-M4 plates begins from (0–24) h in the OmniLog reader; 96-well plate representation in each plate. Each well represents three groups: hypoxia, normoxia (control), and negative control (without cells). Each figure shows the time on the *x*-axis versus OmniLog value on the *y*-axis. PM-M2 and PM-M3 show no change among the groups. PM-M4 shows a biotic reaction in the negative control group with no change in hypoxia and normoxia groups, respectively. The data were analyzed by the OPM package after being extracted from the OmniLog reader.
