# Supplementary material for: Identification of Potential Chemical Substrates as Fuel for Hypoxic Tumors That May Be Linked to Invadopodium Formation in Hypoxia-Induced MDA-MB-231 Breast-Cancer Cell Line
Source: Molecules. 2020 Aug 26;25(17):3876. doi: 10.3390/molecules25173876 (PMC7503683; doi:10.3390/molecules25173876)
Supplement: Supplementary file 1 [file molecules-25-03876-s001.pdf]

Supplementary Materials

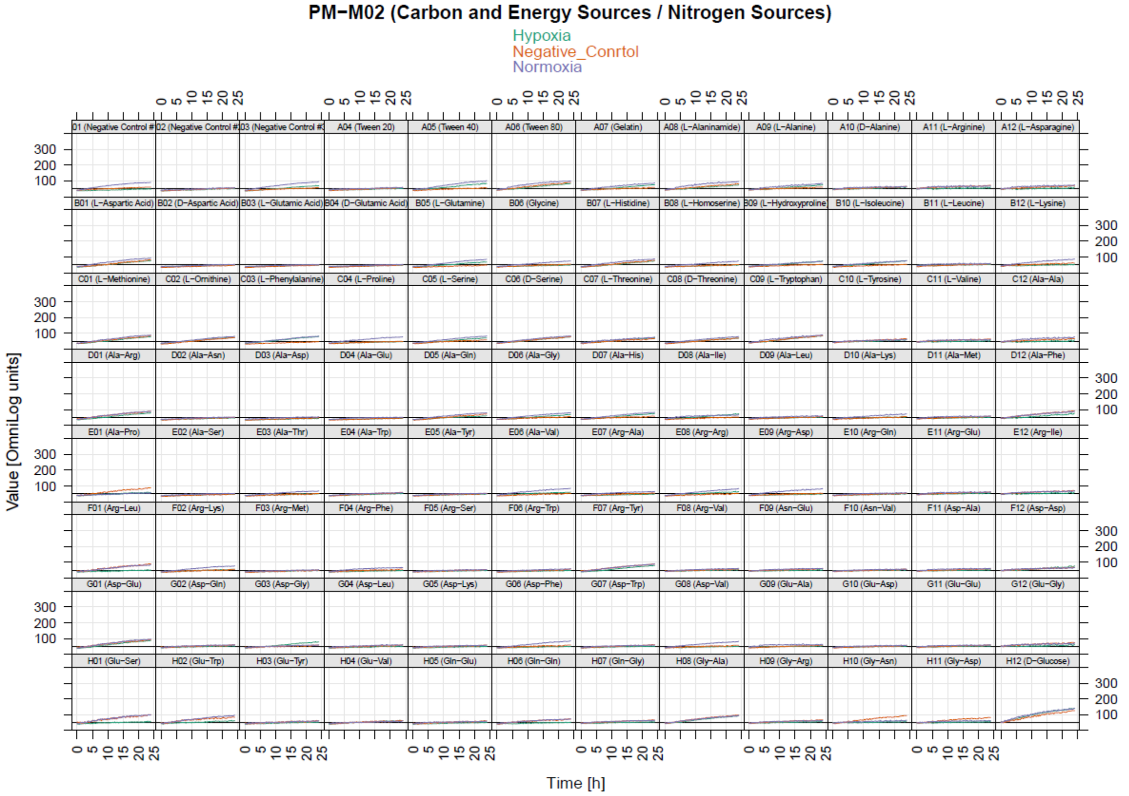

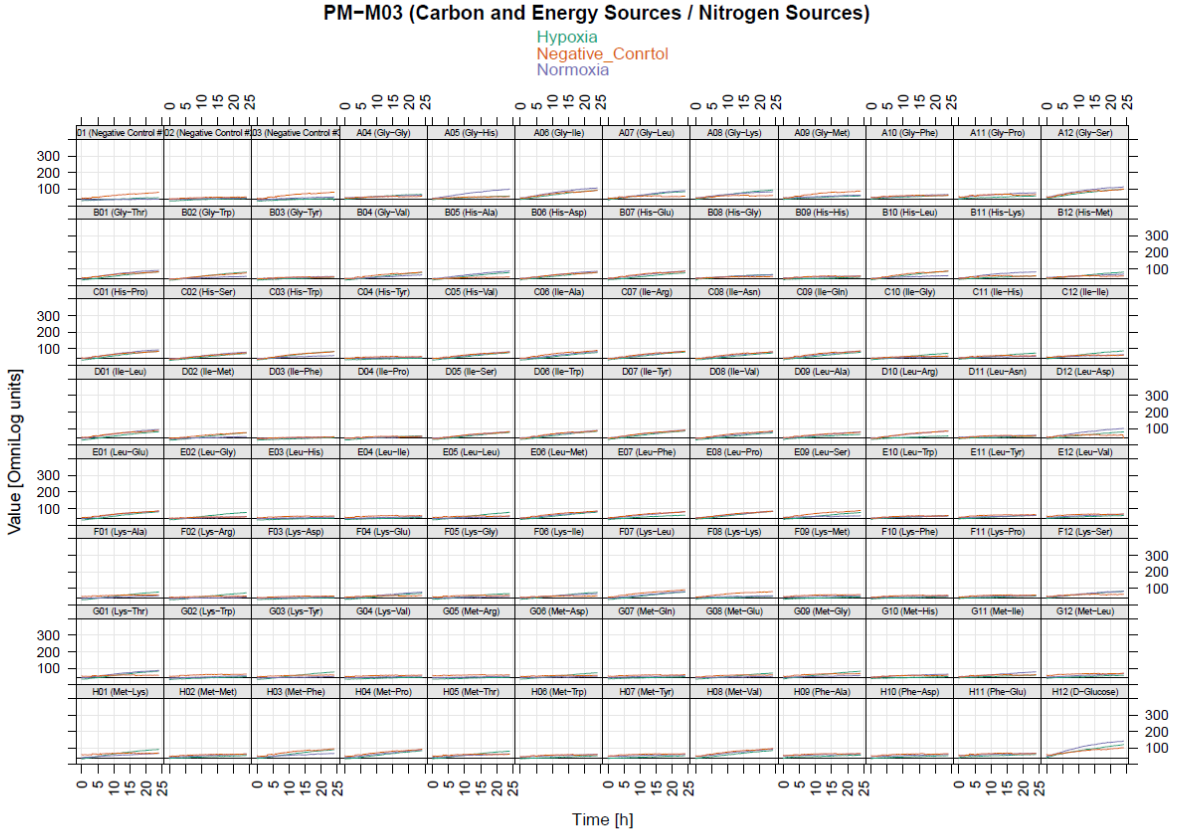

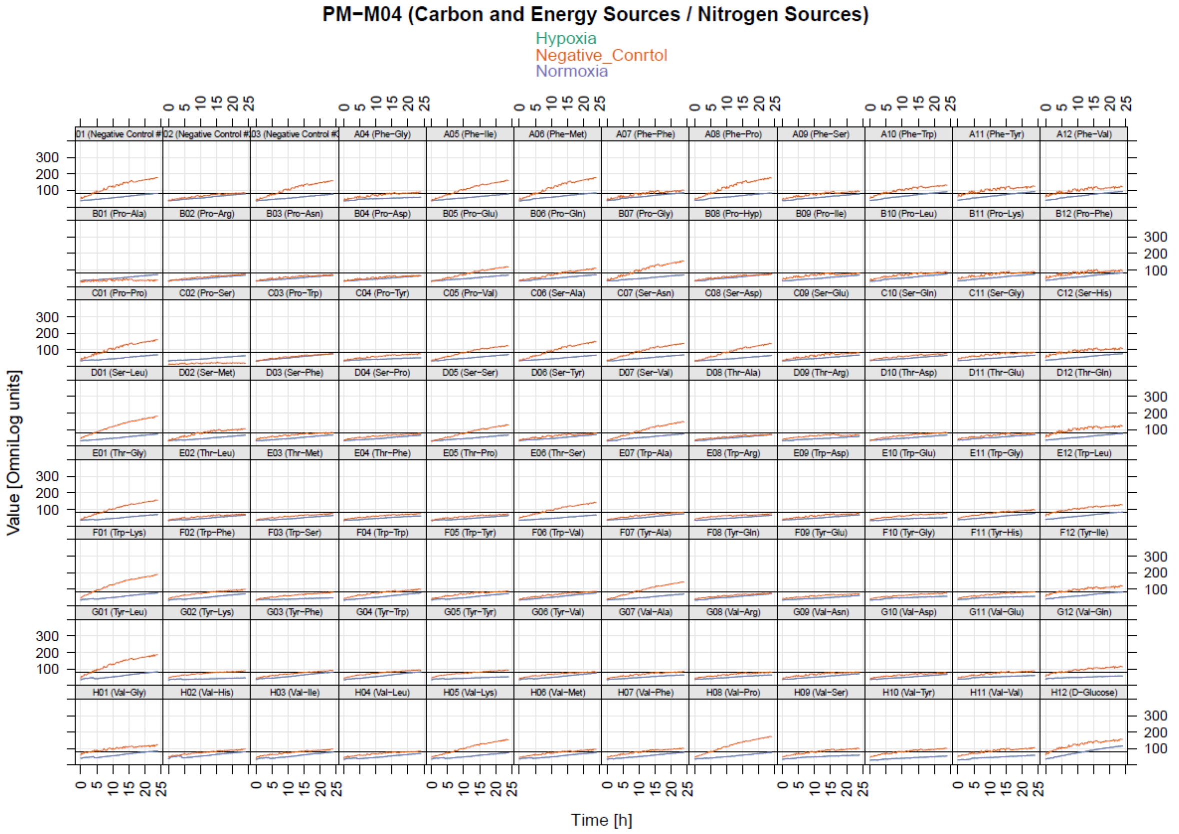

Supplentary data 1. The metabolic fingerprint of phenotype microarray in MDA-MB-231 cell line. Graphical depiction of PM-M2 to PM-M4 plates begins from 0 to 24 h in the OmniLog reader; 96-well plate representation in each plate. Each well represents three groups: hypoxia, normoxia (control), and negative control (without cells). Each figure shows the time on the x-axis versus OmniLog value on the y-axis. PM-M2 and PM-M3 shows no change among the groups. PM-M4 shows a biotic reaction in the negative control group with no change in hypoxia and normoxia groups, respectively. The data were analyzed by OPM package after being extracted from the OmniLog reader.
